# Macrophage Pro-Inflammatory Response to Francisella novicida Infection Is Regulated by SHIP

**DOI:** 10.1371/journal.ppat.0020071

**Published:** 2006-07-21

**Authors:** Kishore V. L Parsa, Latha P Ganesan, Murugesan V. S Rajaram, Mikhail A Gavrilin, Ashwin Balagopal, Nrusingh P Mohapatra, Mark D Wewers, Larry S Schlesinger, John S Gunn, Susheela Tridandapani

**Affiliations:** 1 The Ohio State Biochemistry Program, The Ohio State University, Columbus, Ohio, United States of America; 2 Department of Internal Medicine, The Ohio State University, Columbus, Ohio, United States of America; 3 Department of Molecular Virology, Immunology, and Medical Genetics and Center for Microbial Interface Biology, The Ohio State University, Columbus, Ohio, United States of America; Stanford University, United States of America

## Abstract

*Francisella tularensis,* a Gram-negative facultative intracellular pathogen infecting principally macrophages and monocytes, is the etiological agent of tularemia. Macrophage responses to F. tularensis infection include the production of pro-inflammatory cytokines such as interleukin (IL)-12, which is critical for immunity against infection. Molecular mechanisms regulating production of these inflammatory mediators are poorly understood. Herein we report that the SH2 domain-containing inositol phosphatase (SHIP) is phosphorylated upon infection of primary murine macrophages with the genetically related F. novicida, and negatively regulates F. novicida–induced cytokine production. Analyses of the molecular details revealed that in addition to activating the MAP kinases, F. novicida infection also activated the phosphatidylinositol 3-kinase (PI3K)/Akt pathway in these cells. Interestingly, SHIP-deficient macrophages displayed enhanced Akt activation upon F. novicida infection, suggesting elevated PI3K-dependent activation pathways in absence of SHIP. Inhibition of PI3K/Akt resulted in suppression of F. novicida–induced cytokine production through the inhibition of NFκB. Consistently, macrophages lacking SHIP displayed enhanced NFκB-driven gene transcription, whereas overexpression of SHIP led to decreased NFκB activation. Thus, we propose that SHIP negatively regulates *F. novicida–*induced inflammatory cytokine response by antagonizing the PI3K/Akt pathway and suppressing NFκB-mediated gene transcription. A detailed analysis of phosphoinositide signaling may provide valuable clues for better understanding the pathogenesis of tularemia.

## Synopsis

Francisella tularensis is an intracellular Gram-negative bacterium that causes the disease tularemia. The bacteria infect macrophages and replicate within the host cell. Macrophages respond to the infection by producing pro-inflammatory cytokines in an effort to combat the infection. The intracellular signaling events that are involved in host cell responses are not fully understood. Much less understood are mechanisms that regulate these responses. In this study, the authors define a negative regulatory role for the inositol phosphatase SHIP during *Francisella* infection of mouse macrophages. The study demonstrates that SHIP is activated in infected cells and serves to down-regulate pro-inflammatory cytokine production. The authors have examined the molecular mechanism underlying this negative regulation, and show that SHIP acts upstream of NFκB, an important transcription factor involved in inflammatory cytokine gene transcription, and dampens its activation. This effect of SHIP appears to be mediated through the regulation of the PI3 kinase pathway. This study establishes a novel and critical role for SHIP in the regulation of macrophage inflammatory response to *Francisella* bacteria.

## Introduction

*Francisella tularensis,* causative agent of the zoonotic disease tularemia, is a Gram-negative intracellular pathogen. There are four different subspecies of F. tularensis. The F. tularensis subspecies *tularensis* is the most virulent of the four with a 50% lethal dose (LD_50_) less than 10 colony forming units (CFUs) for humans [1]. Other less virulent subspecies of *F. tularensis* include *novicida, holoarctica,* and *mediasiatica*.

F. tularensis infects primarily monocytes and macrophages. The survival strategy adopted by F. tularensis in the host cell is to avoid phago-lysosomal fusion. After a few hours of phagocytosis of the organism, the membrane of the phagosome ruptures and F. tularensis is released into the host cell cytosol [1,2]. The release of the pathogen into the cytosol requires expression of bacterial proteins such as IglC and MglA [3–5]. On the other hand, bacterial escape is inhibited by interferon-γ (IFNγ) treatment of the cells infected with F. tularensis [6–8].

The host cell responses to F. tularensis infection are not clearly understood. Interleukin (IL)-12 and IFNγ have been reported to be critical for immunity against *Francisella* infection [9]. Although natural killer (NK) cells are thought to be the major source of IFNγ, IL-12 is produced by infected macrophages [10]. Further, IL-12 also appears to strongly induce IFNγ production. Attesting to the importance of IL-12 in immunity against F. tularensis LVS infection, IL-12 knockout animals, or animals treated with IL-12 neutralizing antibodies are unable to clear the bacteria [11]. Macrophages also produce several other pro-inflammatory cytokines/chemokines upon infection [8,12,13]. Intracellular signaling molecules involved in macrophage response to *Francisella* are not well defined. Although pathways involving NFκB and the MAPKs p38 and JNK have been reported to be activated during F. tularensis LVS infection, the exact role of these signaling pathways in macrophage responses is not clear [13].

The Src homology 2 (SH2) domain-containing inositol 5′ phosphatase (SHIP) is a hematopoietic cell-specific phosphatase that negatively regulates phosphatidylinositol 3-kinase (PI3K) pathway by consuming the lipid products of PI3K [14,15]. SHIP is a constitutively active enzyme located in cytosol. Membrane translocation of SHIP is required for access to its substrates. Upon stimulation of immunoreceptors, growth factor/cytokine receptors or toll-like receptors, SHIP translocates to the membrane where it is phosphorylated by membrane-associated Src kinases [16–19]. In addition to the central catalytic domain, SHIP also contains an N-terminal SH2 domain, a C-terminal proline-rich domain, and two NPxY motifs, which can all associate with other cellular signaling proteins. Thus, SHIP mediates its functions by both its catalytic domain and its protein–protein interaction domains. For example, the catalytic domain of SHIP regulates cellular responses by hydrolyzing PI3,4,5P_3_ into PI3,4P_2_, thus antagonizing the PI3K pathway [15]). SHIP is also reported to suppress activation of the Ras pathway, in some cases, by virtue of its interaction with Dok, a Ras GAP activator [20], and/or its interactions with the Ras adapter Shc [21–23].

Herein, we have investigated the role of SHIP in F. novicida–induced cytokine response in murine macrophages. We report that SHIP negatively regulates the production of IL-12, IL-6, and RANTES by F. novicida–infected macrophages. Our current studies indicate that the production of these cytokines requires the activation of the PI3K pathway and involves NFκB activation. These studies also demonstrate that *F. novicida–*induced pro-inflammatory cytokine production is negatively regulated by SHIP by opposing the PI3K pathway and NFκB-driven transcriptional activation. Thus, we conclude that SHIP is a regulator of macrophage innate immune responses to F. novicida infection.

## Results

### *F. novicida–*Induced Pro-Inflammatory Cytokine Response Is Down-Regulated by SHIP

**Figure 1:**
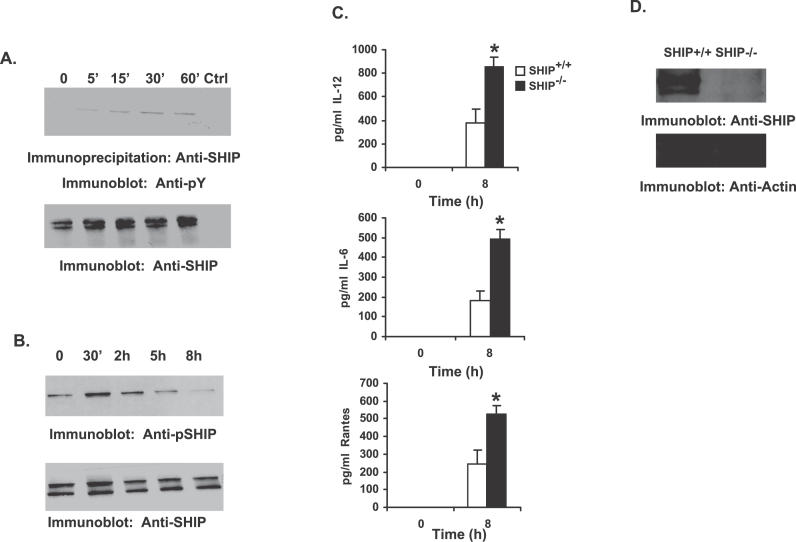
SHIP Down-Regulates Macrophage Pro-Inflammatory Response to F. novicida Infection (A) RAW 264.7 murine macrophage cells were infected with F. novicida (MOI = 100). SHIP immunoprecipitates from uninfected and infected cells were analyzed by Western blotting with anti-phosphotyrosine antibody (Anti-pY; upper panel). The lower panel is a reprobe of the same membrane with anti-SHIP antibody. The lane marked “Ctrl” represents immunoprecipitate with control antibody. (B) BMMs were infected with F. novicida for the times shown in the figure. Protein-matched lysates were separated by SDS/PAGE and analyzed by Western blotting with antibodies specific for phosphorylated SHIP (Anti-pSHIP; upper panel). The lower panel is a reprobe with SHIP antibody (Anti-SHIP). (C) BMMs from SHIP^+/+^ and SHIP^−/−^ littermate mice were infected for 8 h with F. novicida. Cell supernatants from uninfected and infected cells were analyzed by ELISA for IL-12, IL-6, and RANTES. The graph represents mean and standard deviation (SD) of values obtained from three independent experiments. Data were analyzed by paired Student *t* test. An asterisk (*) indicates *p*-value < 0.05. (D) Protein-matched lysates from SHIP^+/+^ and SHIP^−/−^ BMMs were analyzed by Western blotting with SHIP antibody (upper panel). The same membrane was reprobed with actin antibody (Anti-Actin; lower panel).

The inositol phosphatase SHIP plays an important role in regulating macrophage innate immune responses to IgG immune complexes and bacterial products [16,24–29]. Recent studies have indicated that while SHIP negatively regulates TLR2 function [30], it promotes TLR4 function [16,27,29]. SHIP is a constitutively active, cytoplasmic enzyme that must translocate to the membrane where it accesses its substrate PI3,4,5P_3_. Membrane translocation of SHIP is accompanied by tyrosine phosphorylation of SHIP by membrane-associated Src kinases. Thus, tyrosine phosphorylation of SHIP is often used as an indicator of SHIP translocation to the membrane [16]. To address the role of SHIP in F. novicida–stimulated host cell response, RAW 264.7 murine macrophage cells were infected with F. novicida. SHIP proteins were immunoprecipitated from uninfected and infected cells, and analyzed by Western blotting with phosphotyrosine antibody (Figure 1A, upper panel). The same membrane was reprobed with SHIP antibody (lower panel). Results indicate that SHIP is tyrosine phosphorylated in cells infected with *F. novicida,* and that robust phosphorylation of SHIP occurs at the later time points (30 and 60 min post infection). Having determined the time course of SHIP phosphorylation in RAW 264.7 cells, we next tested whether these findings could be validated in primary cells. Here murine bone marrow-derived macrophages (BMMs) were infected with F. novicida for varying times, and phosphorylation of SHIP was analyzed by Western blotting with a phospho-specific SHIP antibody (Figure 1B, upper panel). Results indicated that infection of BMMs by F. novicida induces tyrosine phosphorylation of SHIP (Figure 1B). To ensure equal loading of protein in all lanes, the same membrane was reprobed with anti-SHIP antibody. These results suggest a potential involvement of SHIP in F. novicida–induced macrophage signaling.

In order to probe the functional consequence of SHIP activation, F. novicida–induced cytokine production was compared in BMMs obtained from SHIP^+/+^ and SHIP^−/−^ littermate mice. Thus, SHIP^+/+^ and SHIP^−/−^ BMMs were infected with *F. novicida,* and cell supernatants from uninfected and infected cells were harvested 8 h post infection and analyzed by enzyme-linked immunosorbent assay (ELISA) for IL-12, IL-6, and RANTES. As seen in Figure 1C, SHIP^−/−^ BMMs produced significantly elevated levels of pro-inflammatory mediators compared to their wild-type counterparts. Similar results were obtained with peritoneal macrophages isolated from SHIP^+/+^ and SHIP^−/−^ littermates, and indicate that SHIP-deficient macrophages make more IL-12 and IL-6 (unpublished data; *p* < 0.05). Protein-matched cell lysates from SHIP^+/+^ and SHIP^−/−^ BMMs were analyzed by Western blotting with SHIP antibody to confirm the genotype of these cells (Figure 1D). These data indicate that SHIP is a negative regulator of IL-12, IL-6, and RANTES secretion by *F. novicida–*infected macrophages.

**Figure 2:**
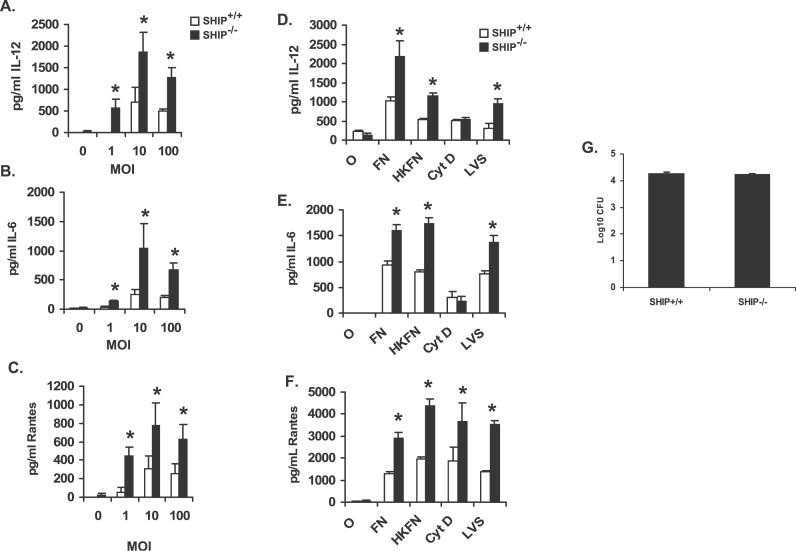
Analysis of SHIP Influence on Macrophage Inflammatory Response to F. novicida Infection (A–C) BMMs from SHIP^+/+^ and SHIP^−/−^ littermate mice were infected at different MOI (indicated in figure) of F. novicida for 8 h. Cytokine levels were measured in uninfected samples (0 h). Cell supernatants from uninfected and infected cells were assayed for IL-12, IL-6, and RANTES by ELISA. (D–F) BMMs from SHIP^+/+^ and SHIP^−/−^ littermate mice were infected with 100 MOI of live or heat-killed F. novicida (FN and HKFN, respectively) or with F. tularensis LVS (LVS) for 8 h. Cyt D represents samples that were treated with cytochalasin D (5 μg/ml) before infection with 100 MOI of live *F. novicida.* Cytokine levels were measured in uninfected samples (0 h). Cell supernatants from uninfected and infected cells were analyzed by ELISA for IL-12, IL-6, and RANTES. (G) BMMs from SHIP ^+/+^ and SHIP^−/−^ littermate mice were infected with 100 MOI of F. novicida for 2 h, then treated with gentamicin (50 μg/ml), lysed in 0.1% SDS and appropriate dilutions of the lysates were plated on Chocolate II agar plates for enumeration of CFUs. All graphs represents mean and SD of values obtained from three independent experiments. Data were analyzed by paired Student *t* test. An asterisk (*) indicates *p*-value < 0.05.

The regulatory influence of SHIP on F. novicida–induced IL-12, IL-6, and RANTES was also observed at different multiplicities of infection (MOIs; Figure 2A–2C), suggesting that the negative regulatory effect of SHIP is independent of bacterial numbers infecting the macrophages. In addition, the viability of the bacteria is not essential for the negative influence of SHIP as macrophages infected with heat-killed bacteria displayed similar cytokine responses as those infected with live bacteria (Figure 2D–2F). Likewise, SHIP also down-regulated F. tularensis LVS–induced IL-12, IL-6, and RANTES production (Figure 2D–2F).

We next examined whether internalization of bacteria is a requisite for the down-regulatory influence of SHIP on F. novicida–induced cytokine/chemokine production. For this, cells were treated with cytochalasin D, an actin polymerization inhibitor, prior to infection, and cell supernatants were assayed for the secretion of IL-12, IL-6, and RANTES by ELISA. The results are shown in Figure 2D–2F. The release of RANTES and the negative influence of SHIP on RANTES production were not influenced by failure of internalization. However, treatment of either SHIP^+/+^ or SHIP^−/−^ BMM with cytochalasin D significantly suppressed the release of IL-12 and IL-6, suggesting that either internalization of bacteria or actin cytoskeletal rearrangements are essential for the secretion of these two cytokines. Failure of internalization of F. novicida did not influence the activation of either the MAPK or the PI3K/Akt pathways (unpublished data), suggesting that surface contact of *Francisella* bacteria is sufficient to trigger the signaling response.

We then examined whether the increased production of cytokine mediators by SHIP^−/−^ macrophages is due to enhanced uptake of F. novicida. The uptake of bacteria was determined by CFU assays, and the results are shown in Figure 2G. The uptake of F. novicida by SHIP^+/+^ and SHIP^−/−^ BMM was equivalent. These findings are also supported by transmission electron microscopy (TEM) analysis (SHIP^+/+^ and SHIP^−/−^ macrophages ingested 2.95 and 3.2 bacteria/cell, respectively).

### PI3K/Akt Pathway Is Activated upon F. novicida Infection of Macrophages

**Figure 3:**
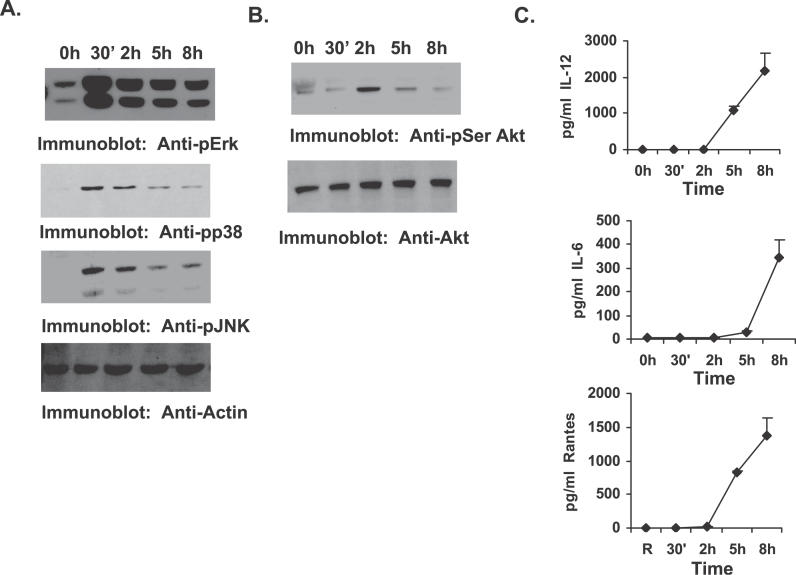
PI3K/Akt Pathway Is Activated upon F. novicida Infection BMMs were infected with F. novicida for the times shown in the figure. (A) Protein-matched lysates were analyzed by Western blotting with phospho-specific antibodies to Erk (Anti-pErk), p38 (Anti-pp38), and JNK (Anti-pJNK). The membranes were reprobed with actin antibody (Anti-Actin) to ensure equal loading of protein in all lanes. (B) Protein-matched lysates were analyzed by Western blotting with phospho-specific antibody to Akt (Anti-pSer Akt; upper panel). The lower panel is a reprobe of the same membrane with total Akt antibody (Anti-Akt). (C) Cell supernatants from the same experiments were assayed by ELISA for IL-12, IL-6, and RANTES. The graph represents mean and SD of values obtained from three independent experiments.

To examine the mechanism by which SHIP down-regulates pro-inflammatory cytokine production by *F. novicida–*infected macrophages, cell signaling events activated in infected macrophages were first analyzed. Here, murine BMMs were infected with F. novicida for varying time points, and the following analyses were performed with the cell lysates and cell supernatants. First, activation of the MAP kinases Erk, p38, and JNK was analyzed by Western blotting protein-matched lysates with phospho-specific antibodies (Figure 3A). All membranes were reprobed with antibody to actin to ensure equal loading of protein in all lanes. Results indicated that all three MAPKs were activated in response to F. novicida infection as previously reported [13]. The phosphorylation levels of MAPKs peaked at 30 min and gradually diminished, but were still persistent even at 8 h post infection. Second, we examined the activation of the PI3K pathway by analyzing phosphorylation status of the PI3K-dependent serine/threonine kinase Akt. The results shown in Figure 3B indicate that the PI3K/Akt pathway is also activated during F. novicida infection. Finally, cell supernatants were analyzed for pro-inflammatory cytokines by ELISA. As seen in Figure 3C, BMMs infected with F. novicida produced significant amounts of the pro-inflammatory mediators IL-12, IL-6, and RANTES, which became detectable at about 5 h post infection.

### SHIP Regulates F. novicida–Induced Activation of the PI3K/Akt Pathway

**Figure 4:**
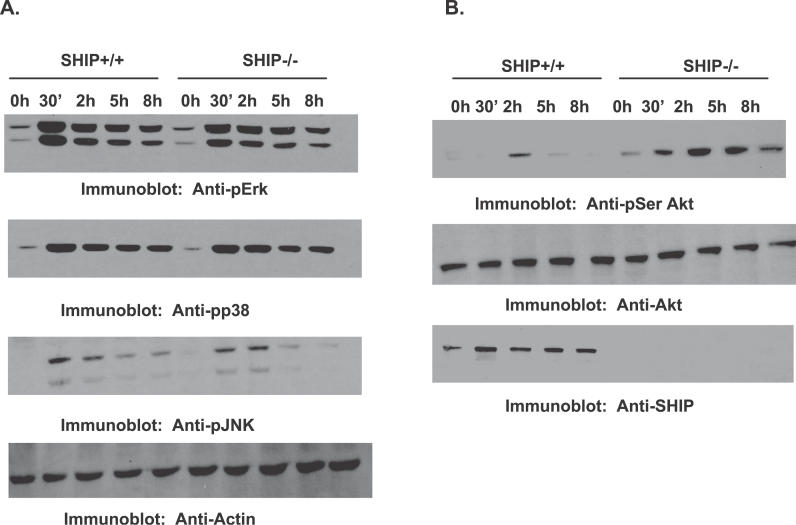
SHIP Negatively Regulates F. novicida–Induced Akt Activation BMMs from SHIP^+/+^ and SHIP^−/−^ littermate mice were infected for the times indicated in the figure. (A) Protein-matched lysates were probed with antibodies specific for phosphorylated Erk (Anti-pErk), p38 (Anti-pp38), and JNK (Anti-pJNK). All membranes were reprobed with actin antibody (Anti-Actin). (B) Protein-matched lysates were probed with phospho-specific antibody for Akt (Anti-pSer Akt; upper panel). The membranes were reprobed with total Akt antibody (Anti-Akt; middle panel), and with SHIP antibody (Anti-SHIP; lower panel). These data are representative of three independent experiments.

Having identified cellular signaling events activated by F. novicida infection, we next examined the influence of SHIP on these events. For this, BMMs from SHIP^+/+^ and SHIP^−/−^ littermate mice were infected with F. novicida for the times indicated in Figure 4, and activation of MAPKs and Akt was analyzed. Infection by F. novicida induced robust phosphorylation of Erk1/2, JNK, p38, and Akt. However, SHIP^+/+^ and SHIP^−/−^ BMMs displayed no significant differences in the activation of the MAPKs at any of the time points tested. On the other hand, SHIP^−/−^ BMMs showed enhanced phosphorylation of Akt compared to SHIP^+/+^ BMMs. These results indicate that although SHIP may not regulate F. novicida–induced activation of the MAPKs, SHIP down-regulates activation of Akt. Together, these data suggest that the PI3K/Akt pathway may play a critical role in F. novicida–induced macrophage pro-inflammatory responses.

### Influence of PI3K Pathway on F. novicida–Induced Macrophage Inflammatory Response

**Figure 5:**
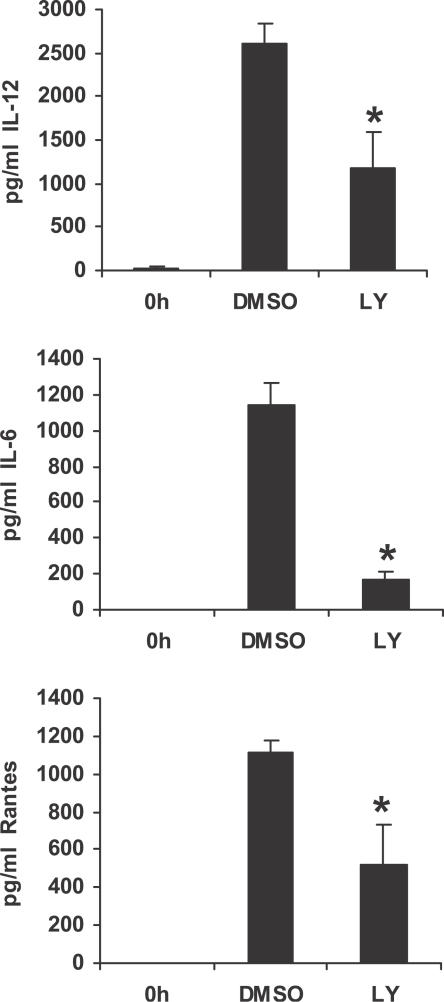
PI3K Activation Promotes F. novicida–Induced Macrophage Inflammatory Response BMMs were pretreated for 30 min with PI3K inhibitor LY294002 (LY; 20 μM), or with vehicle control (DMSO), and subsequently infected with F. novicida. Cell supernatants were harvested 8 h post infection and assayed by ELISA for IL-12, IL-6, and RANTES. Cytokine levels were measured in uninfected samples (0 h). The graphs represent mean and SD of values obtained from three independent experiments. Data were analyzed by paired Student *t* test. An asterisk (*) indicates *p*-value < 0.05.

A role for the PI3K/Akt pathway in macrophage response to F. novicida infection has thus far not been described. The above observations of enhanced cytokine production and enhanced activation of Akt in SHIP^−/−^ BMMs suggest a potential role for the PI3K/Akt pathway in F. novicida–induced inflammatory response. To test this notion, we next treated F. novicida infected cells with the pharmacologic inhibitor of PI3K LY294002, and monitored the release of IL-12, IL-6, and RANTES by ELISA. The results are displayed in Figure 5. Inhibition of the PI3K pathway significantly decreased the production of IL-12 (*p* < 0.009), IL-6 (*p* < 0.003), and RANTES (*p* < 0.03) by F. novicida–infected BMMs, indicating that the PI3K pathway is involved in the production of pro-inflammatory mediators in response to F. novicida infection.

### The PI3K/Akt Pathway Promotes Macrophage Inflammatory Response to F. novicida through Its Influence on Downstream NFκB Activation

**Figure 6:**
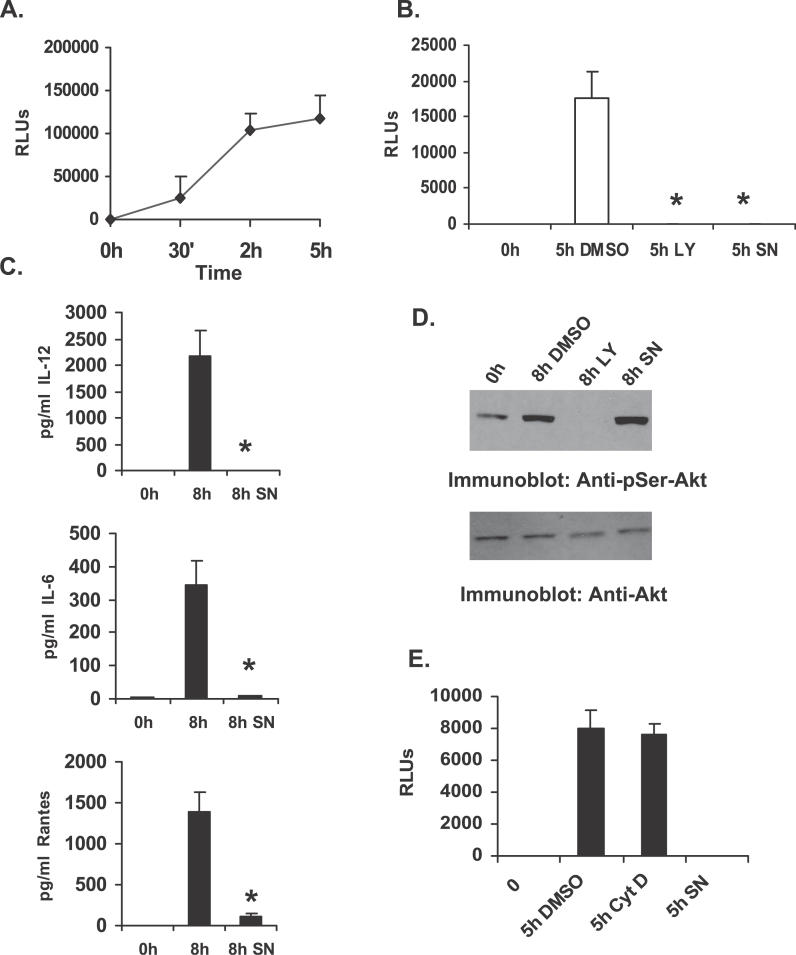
PI3K Promotes F. novicida–Induced Inflammatory Response through Its Influence on NFκB (A) RAW 264.7 cells transfected with plasmid encoding the *luciferase* gene driven by an NFκB binding element (NFκB-luc). Transfectants were infected with F. novicida for varying times and analyzed for the luciferase activity as a measure of NFκB activation. RLUs, relative light units. (B) RAW 264.7 cells transfected with NFκB-luc plasmid were pretreated with either a PI3K inhibitor LY294002 (LY), a NFκB inhibitor SN50 (SN; 75 μg/ml), or with vehicle control (DMSO), and subsequently infected with F. novicida. At 5 h post infection, cells were lysed and assayed for luciferase activity. RLUs, relative light units. (C) BMMs were pretreated for 30 min with either DMSO (middle bar) or the NFκB inhibitor SN50 (SN), and subsequently infected with F. novicida. Cell supernatants were harvested 8 h post infection and assayed for IL-12, IL-6, and RANTES by ELISA. Cytokine levels were measured in uninfected samples (0 h). The graph represents mean and SD of values obtained from three experiments. Data were analyzed by paired Student *t* test. An asterisk (*) indicates *p*-value < 0.05. (D) Protein-matched lysates from BMMs pretreated with either DMSO or with inhibitors of PI3K (LY) or NFκB (SN) and infected with F. novicida were analyzed by Western blotting with phospho-specific antibody to Akt (Anti-pSer-Akt; upper panel). The lower panel is a reprobe of the same membrane with Akt antibody (Anti-Akt). (E) RAW 264.7 cells transfected with NFκB-luc plasmid were pretreated with either cytochalasin D (Cyt D), SN50 (SN), or with vehicle control (DMSO), and subsequently infected with F. novicida. At 5 h post infection, cells were lysed and assayed for luciferase activity. The data are representative of three independent experiments.

We next examined the molecular mechanism by which PI3K/Akt influences F. novicida–induced cytokine response. It has been previously reported that F. novicida induces the activation of NFκB, and that activation of this transcription factor may play an important role in the secretion of pro-inflammatory cytokines. Hence we wanted to test the involvement of NFκB in the secretion of IL-12, IL-6, and RANTES in response to F. novicida infection, and the influence of PI3K on NFκB activation. For this, we first transiently transfected RAW 264.7 cells with a construct encoding luciferase reporter gene under the influence of NFκB binding (NFκB-luciferase construct). Twelve hours post transfection, cells were infected with F. novicida for the times indicated in Figure 6A, and luciferase activity in the cell lysate was determined. Results indicated that NFκB activation is induced in response to F. novicida infection.

Second, to test whether PI3K influences F. novicida–induced activation of NFκB, transfected cells were treated with LY294002, or an inhibitor of NFκB SN50, and expression of luciferase enzyme in response to F. novicida infection was measured. The results are shown in Figure 6B. Inhibition of PI3K with LY294002 significantly decreased expression of the NFκB-driven reporter gene in response to F. novicida infection (*p* < 0.0001), suggesting that the activation of PI3K pathway is necessary for induction of NFκB activation.

Finally, inhibition of NFκB, using the pharmacologic inhibitor SN50, in murine BMMs infected with F. novicida resulted in significant inhibition of IL-12 (*p* < 0.009), IL-6 (*p* < 0.007), and RANTES (*p* < 0.02) production (Figure 6C). The Western blots shown in Figure 6D demonstrate that although treatment of cells with the PI3K inhibitor completely abrogated downstream Akt phosphorylation, Akt phosphorylation was not influenced by the NFκB inhibitor SN50, indicating that SN50 inhibition has occurred downstream of Akt. Collectively, these data provide evidence that PI3K influences F. novicida–induced macrophage pro-inflammatory response through its influence on NFκB activation. Of note, the activation of NFκB is unaffected in macrophages pre-treated with cytochalasin D, suggesting that internalization of F. novicida is not required (Figure 6E).

### SHIP Negatively Regulates F. novicida–Induced NFκB Activation

**Figure 7:**
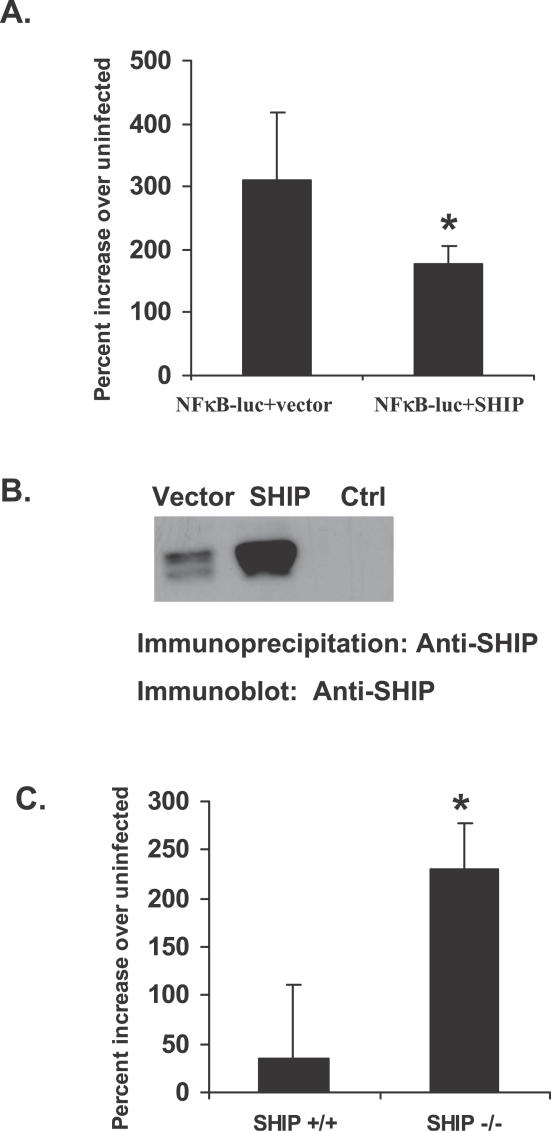
SHIP Negatively Regulates F. novicida–Induced NFκB Activation (A) RAW 264.7 cells were transfected with NFκB-luc plasmid along with either empty vector or plasmid encoding SHIP. Transfectants were infected with F. novicida and assayed for luciferase activity 5 h post infection. Data were analyzed by Student *t* test. An asterisk (*) indicates *p*-value < 0.05. (B) SHIP immunoprecipitates from the RAW 264.7 transfectants described in Figure 7A were analyzed by Western blotting for overexpression of SHIP. Ctrl indicates immunoprecipitation with normal rabbit serum. (C) SHIP^+/+^ and SHIP^−/−^ BMMs were transfected with NFκB-luc plasmid. Transfectants were infected with F. novicida and assayed for luciferase activity. Graphs represent values from three independent experiments. Data were analyzed by Student *t* test. An asterisk (*) indicates *p*-value < 0.05.

Requirement of PI3K activity for F. novicida–induced NFκB activation suggested that SHIP may regulate F. novicida–stimulated NFκB activation. To test this prediction, RAW 264.7 cells were transfected with NFκB–luciferase construct alone along with either a construct encoding SHIP or the corresponding empty vector. Transfectants were then infected with *F. novicida,* and luciferase activity in the cell lysates was measured as an indicator of NFκB activation. As expected, overexpression of SHIP significantly (*p* < 0.04) suppressed NFκB-dependent reporter gene expression (Figure 7A). Overexpression of SHIP in the transfected cells was confirmed by Western blotting SHIP immunoprecipitates with SHIP antibody (Figure 7B).

As an additional approach, murine BMMs obtained from SHIP ^+/+^ and SHIP ^−/−^ mice were transiently transfected with the NFκB–luciferase construct. Transfection of BMMs using the amaxa Nucleofector (Solution T, program T-20; amaxa biosystems, Cologne, Germany) yielded comparable transfection efficiency for both SHIP^+/+^ and SHIP^−/−^ cells, as determined with plasmid encoding EGFP (enhanced green fluorescent protein; unpublished data). Transfected BMMs were subsequently infected with F. novicida and assayed for luciferase activity. BMMs lacking SHIP displayed significantly higher levels (*p* < 0.05) of luciferase activity than the SHIP^+/+^ BMMs, indicating that SHIP negatively regulates F. novicida–induced activation of NFκB (Figure 7C).

### SHIP Positively Regulates F. novicida–Induced IL-10 Production

**Figure 8:**
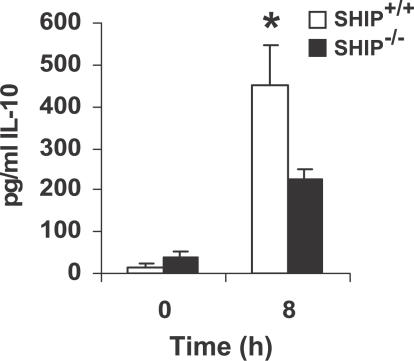
SHIP Influence on Macrophage IL-10 Response to F. novicida BMMs from SHIP^+/+^ and SHIP^−/−^ littermate mice were infected for 8 h with *F. novicida.* Cell supernatants from uninfected and infected cells were analyzed by ELISA for IL-10. The graph represents mean and SD of values obtained from three independent experiments. Data were analyzed by paired Student *t* test. An asterisk (*) indicates *p*-value < 0.05.

To determine whether SHIP regulates the production of anti-inflammatory cytokines by infected macrophages, we next measured IL-10 production. Here SHIP^+/+^ and SHIP^−/−^ BMMs were infected with F. novicida for 8 h. Cell supernatants from uninfected and infected cells were analyzed by ELISA for IL-10. The results shown in Figure 8 demonstrate that SHIP^+/+^ BMMs produce significantly higher levels of IL-10 than the SHIP^−/−^ cells.

## Discussion

The molecular details of host response to F. tularensis infection are not clearly understood. Both innate and adaptive immune responses provide protection against this infection. Early protection against F. tularensis is dependent upon the production of IFNγ, TNF-α, and IL-12, all of which are produced within a day after infection [7,9,31,32]. To date, regulatory mechanisms controlling the production of pro-inflammatory molecules have not been established. SHIP is a critical regulator of hematopoietic cell functions; hence we have investigated the role of SHIP in F. novicida–induced inflammatory response. Our data indicate that SHIP is a negative regulator of *Francisella*-induced IL-12, IL-6, and RANTES production by BMMs.

SHIP has been shown to influence hematopoietic cell functions through its inhibitory effect on the PI3K pathway as well as the MAPK pathways [15]. Since previous studies and our current studies have demonstrated the activation of MAPKs in *Francisella*-infected macrophages, we examined whether SHIP influenced activation of the MAPKs. However, our studies indicate that the molecular mechanism by which SHIP influences macrophage response to *Francisella* does not involve inhibition of the MAPKs. Thus, we did not observe any significant differences in the activation levels of MAPKs at any of the time points tested, ranging from 5 min to 8 h (Figure 4A and unpublished data). However, we observed significantly elevated Akt activation in SHIP-deficient macrophages infected with *Francisella*. The hyperactivation of Akt in SHIP-deficient macrophages suggested that the PI3K pathway may play a role in macrophage response to *Francisella*. Consistently, inhibition of PI3K attenuated F. novicida–induced activation of NFκB and the subsequent production of pro-inflammatory cytokines.

Activation of the PI3K/Akt pathway by *Francisella* has not been previously reported. However, other intracellular pathogens such as Salmonella enterica have been shown to modulate host cell phosphoinositide pathways and downstream Akt activation [33]. In the latter case, the functional consequence of phosphoinositide signaling is unclear. Interestingly, pharmacologic inhibition of PI3K failed to prevent invasion of *Salmonella,* suggesting that PI3K may not play a role in internalization of some intracellular pathogens, which use a type III secretion-mediated mechanism to gain entry into the host cell [33,34]. This is in contrast to receptor-mediated phagocytosis in which PI3K activation is critical.

Macrophage receptors that sense *Francisella* and mediate phagocytosis are just being defined [35], but the linkages between receptors and intracellular signaling pathways are essentially unknown. Earlier studies suggested that, since there is a lack of macrophage inflammatory response to the LPS of *Francisella,* it is unlikely that TLR4 is involved [36–38]. Other studies propose that TLR2 may be important [39]. Recent work also suggests a role for intracellular recognition of the pathogen [39]. The role of SHIP in pro-inflammatory cytokine response elicited by TLR4 or TLR2 engagement has been studied. Thus, studies by Strassheim et al. demonstrated that SHIP negatively regulates TLR2 signaling [30]. In contrast, the presence of SHIP appears to enhance TLR4-induced inflammatory response. In an earlier study, we have demonstrated that SHIP-deficient macrophages are hyporesponsive to TLR4 engagement compared to their wild-type counterparts [16]. These findings are supported by recent reports by Rauh et al. [27,29]. Although the latter group earlier reported a negative regulatory role for SHIP [40], these findings were later attributed by the same group to in vitro culture conditions of macrophages in their study [29]. Our current findings that SHIP negatively regulates *Francisella*-induced inflammatory response are consistent with a role for TLR2 in *Francisella*-induced signaling. Additional studies are required to test this notion.

SHIP is a cytosolic enzyme that must undergo membrane translocation to access its lipid substrates. Previous reports indicate that the N-terminal SH2 domain of SHIP is critical for this translocation under certain stimulation conditions, whereas the C-terminal region may be more important under other conditions [15]. Indeed, the C-terminal proline-rich region of SHIP has been shown to be required for stabilization of SHIP at the membrane [41,42]. Additional studies are required to understand the mechanism by which SHIP translocates to the membrane in response to *Francisella* infection. A thorough understanding of the role of SHIP in *Francisella-*induced signaling events may shed light on regulatory mechanisms controlling the production of inflammatory mediators that are essential for protection against *Francisella* infection.

The production of pro-inflammatory cytokines such as IL-12 by monocytes/macrophages contributes further to immunity against *Francisella* infection by augmenting NK cell IFNγ production. In a recent report, we have demonstrated that production of IFNγ by NK cells in response to stimulation by monokines (IL-12, IL-15, and IL-18) is augmented in the absence of SHIP [43,44]. Thus, it is conceivable that SHIP not only regulates macrophage responses to *Francisella* infection, but also the subsequent NK cell responses, thereby regulating overall defense against intracellular pathogens.

In conclusion, this study unravels novel roles for PI3K and SHIP in regulating intracellular signaling events involved in macrophage innate immune response to *Francisella* infection.

## Materials and Methods

### Cells, antibodies, and reagents.

RAW 264.7 murine macrophage cells were obtained from ATCC (Washington, D. C., United States) and maintained in RPMI with 3.5% heat-inactivated fetal bovine serum (FBS). Antibodies specific for phospho-Erk, phospho-JNK, phospho-SHIP, phospho-Akt, and phospho-p38 were purchased from Cell Signaling Technology (Beverly, Massachusetts, United States). Actin, phosphotyrosine, and Akt antibodies were from Santa Cruz Biotechnology (Santa Cruz, California, United States). Rabbit polyclonal SHIP antibody was a generous gift from Dr. K. Mark Coggeshall (Oklahoma Medical Research Foundation, Oklahoma City, Oklahoma, United States). F. novicida U112 (JSG1819) was used in all experiments. Bacteria were grown on Chocolate II agar plates at 37 °C.

### Culture of murine bone marrow macrophages.

SHIP^+/−^ animals were generously provided by Dr. G. Krystal (BC Cancer Agency, Vancouver, British Columbia, Canada). Heterozygotes were bred to obtain SHIP^+/+^ and SHIP^−/−^ mice. BMMs were derived from these animals as previously described [16]. Briefly, bone marrow cells were cultured in RPMI containing 10% fetal bovine serum plus 10 μg/ml polymixin B and supplemented with 20 ng/ml CSF-1 for 7 d before they were used in experiments. BMMs derived in this manner were more than 99% positive for Mac-1, as determined by flow cytometry.

### Cell stimulation, lysis, and Western blotting.

BMMs were plated in six-well culture dishes. Macrophages were infected either with F. novicida (at 1. 10, or 100 MOI) or with F. tularensis LVS (100 MOI) that were scraped from Chocolate II agar plates, resuspended, and diluted in RPMI. For some experiments, bacteria were killed by heat at 98 °C for 10 min prior to adding to the macrophages. To prevent internalization of bacteria, cells were treated with 5 μg/ml of cytochalasin D for 30 min at 37 °C and 5% C0_2_ prior to infection. Uninfected and infected cells were lysed in TN1 buffer (50 mM Tris [pH 8.0], 10 mM EDTA, 10m M Na_4_P_2_O_7_, 10 mM NaF, 1% Triton-X 100, 125 mM NaCl, 10 mM Na_3_VO_4_, 10 μg/ml each aprotinin and leupeptin). Postnuclear lysates were boiled in Laemmli Sample Buffer and were separated by SDS/PAGE, transferred to nitrocellulose filters, probed with the antibody of interest, and developed by enhanced chemiluminescence (ECL).

### CFU assays.

SHIP^+/+^ and SHIP^−/−^ BMMs were infected with F. novicida (100 MOI). Two hours post infection, cells were washed two times and incubated with 50 μg/ml of gentamicin for 30 min at 37 °C and 5% CO_2_. The cells were subsequently washed twice and lysed in 0.1% SDS for 5 min. Immediately, 10-fold serial dilutions were made, and appropriate dilutions were plated on Chocolate II agar plates. Assays were performed in triplicate for each test group.

Transmission electron microscopy (TEM): SHIP^+/+^ and SHIP^−/−^ BMMs were infected with *F. novicida,* and 2 h post infection, the cells were washed, fixed, and prepared for TEM analysis as described previously. Bacterial count in 20 SHIP^+/+^ and SHIP^−/−^ BMMs was assessed and averaged [45].

### ELISA measurement of cytokine production.

BMMs were infected with F. novicida for varying time points. Cell supernatants were harvested, centrifuged to remove dead cells, and analyzed by ELISA using cytokine-specific kits from R & D Systems (Minneapolis, Minnesota, United States). Data were analyzed using a paired Student *t*-test. A *p*-value < 0.05 was considered as significant.

### Transfection and luciferase assays.

BMMs and RAW 264.7 cells were transfected with the appropriate plasmid DNA using the Amaxa Nucleofector apparatus (amaxa biosystems) as previously described [46,47]. Briefly, 5 × 10^6^ cells were resuspended in 100 μl Nucelofector Solution (T for BMMs and V for RAW 264.7 cells), and were nucelofected with 1 μg of NFκB-luc alone or with 5 μg of empty vector or plasmid encoding WT-SHIP. Immediately post nucleofection, 500 μl of pre-warmed RPMI was added to the transfection mix before transferring to 12-well plates containing 1.5-ml pre-warmed RPMI per well. Plates were incubated for 12 h at 37 °C. Transfected cells were either left uninfected or were infected for 5 h with F. novicida. Cells were lysed in 100 μl of Luciferase Cell Culture Lysis Reagent (Promega, Madison, Wisconsin, United States). Luciferase activity was then measured using Luciferase Assay Reagent (Promega), as previously described [24].

## Abbreviations

BMM: bone marrow-derived macrophage
CFU: colony forming unit
ELISA: enzyme-linked immunosorbent assay
IFNγ: interferon-γ
IL: interleukin
MOI: multiplicity of infection
NK: natural killer
PI3K: phosphatidylinositol 3-kinase
SD: standard deviation
SH2: Src homology 2
SHIP: SH2 domain-containing inositol phosphatase
